# Correlation between cerebrospinal fluid and plasma neurofilament light protein in treated HIV infection: results from the COBRA study

**DOI:** 10.1007/s13365-021-01026-3

**Published:** 2021-12-07

**Authors:** Jasmini Alagaratnam, Davide De Francesco, Henrik Zetterberg, Amanda Heslegrave, Jamie Toombs, Neeltje A. Kootstra, Jonathan Underwood, Magnus Gisslen, Peter Reiss, Sarah Fidler, Caroline A. Sabin, Alan Winston

**Affiliations:** 1 Department of Infectious Disease, Faculty of Medicine, Imperial College London, London, UK; 2 Department of Genitourinary Medicine &, HIV, St Mary’s Hospital, Imperial College Healthcare NHS Trust, London, UK; 3 Institute for Global Health, University College London, London, UK; 4 UK Dementia Research Institute at University College London, London, UK; 5 Department of Neurodegenerative Disease, UCL Institute of Neurology, Queen Square, University College London, London, UK; 6 Department of Psychiatry and Neurochemistry, Institute of Neuroscience and Physiology, the Sahlgrenska Academy at the University of Gothenburg, Mölndal, Sweden; 7 Clinical Neurochemistry Laboratory, Sahlgrenska University Hospital, Mölndal, Sweden; 8 Department of Experimental Immunology, Amsterdam UMC, University of Amsterdam, Amsterdam, The Netherlands; 9 Division of Infection and Immunity, Cardiff University, Cardiff, UK; 10 Department of Infectious Diseases, Cardiff and Vale University Health Board, Cardiff, UK; 11 Department of Infectious Diseases, Institute of Biomedicine, Sahlgrenska Academy, University of Gothenburg, Gothenburg, Sweden; 12 Department of Infectious Diseases, Region Västra Götaland, Sahlgrenska University Hospital, Gothenburg, Sweden; 13 Department of Global Health, Amsterdam University Medical Centers, University of Amsterdam, Amsterdam, The Netherlands; 14 Amsterdam Institute for Global Health and Development, Amsterdam, The Netherlands; 15 Stichting HIV Monitoring, Amsterdam, The Netherlands

**Keywords:** HIV, HIV-1, Central nervous system diseases, Axons, Neurofilament proteins

## Abstract

Cerebrospinal fluid (CSF) neurofilament light protein (NfL) is a marker of central nervous system neuro-axonal injury. A novel, ultra-sensitive assay can determine plasma NfL. In untreated people-with-HIV (PWH), CSF and plasma NfL are strongly correlated. We aimed to assess this correlation in PWH on suppressive antiretroviral treatment (ART) and lifestyle-similar HIV-negative individuals enrolled into the COmorBidity in Relation to AIDS (COBRA) study. Differences in paired CSF (sandwich ELISA, UmanDiagnostics) and plasma (Simoa digital immunoassay, Quanterix™) NfL between PWH and HIV-negative participants were tested using Wilcoxon’s test; associations were assessed using Pearson’s correlation. CSF and plasma NfL, standardised to *Z*-scores, were included as dependent variables in linear regression models to identify factors independently associated with values in PWH and HIV-negative participants. Overall, 132 PWH (all with plasma HIV RNA < 50 copies/mL) and 79 HIV-negative participants were included. Neither CSF (median 570 vs 568 pg/mL, *p* = 0.37) nor plasma (median 10.7 vs 9.9 pg/mL, *p* = 0.15) NfL differed significantly between PWH and HIV-negative participants, respectively. CSF and plasma NfL correlated moderately, with no significant difference by HIV status (PWH: rho = 0.52; HIV-negative participants: rho = 0.47, *p* (interaction) = 0.63). In multivariable regression analysis, higher CSF NfL *Z*-score was statistically significantly associated with older age and higher CSF protein, and higher plasma NfL *Z*-score with older age, higher serum creatinine and lower bodyweight. In conclusion, in PWH on ART, the correlation between CSF and plasma NfL is moderate and similar to that observed in lifestyle-similar HIV-negative individuals. Consideration of renal function and bodyweight may be required when utilising plasma NfL.

## Introduction

Cerebrospinal fluid (CSF) neurofilament light chain protein (NfL) is well-established as a sensitive biomarker of active central nervous system (CNS) neuro-axonal injury (Gaetani et al. 2019; Khalil et al. 2018; Olsson et al. 2019). The neurofilaments form a major structural component of myelinated axons and maintain the structural and functional integrity of axons (Gaetani et al. 2019). Injured axons leak neurofilament protein into the CSF, proportional to the severity of the injury(Gaetani et al. 2019). Elevated CSF NfL concentrations are well-documented in many neurological disorders (Bridel et al. 2019; Gaetani et al. 2019; Khalil et al. 2018; Zetterberg 2016). In HIV-disease, CSF NfL is significantly elevated in individuals with HIV-associated dementia (Abdulle et al. 2007; Gisslén et al. 2007; Krut et al. 2014; Yilmaz et al. 2017), but frequently near-normalise following antiretroviral treatment (ART) (Abdulle et al. 2007; Krut et al. 2014; Mellgren et al. 2007). Elevated CSF NfL concentrations can also be detected in neuroasymptomatic people with HIV (PWH) not on ART, especially in those with low CD4^+^ counts (Krut et al. 2014; Peterson et al. 2014).

The invasive nature of CSF collection via lumbar puncture significantly limits its widespread use. A novel ultra-sensitive assay can determine plasma NfL (Gisslén et al. 2015; Hendricks et al. 2019), which removes the barriers faced by CSF collection and allows more frequent measurement.

Early studies suggest that CSF strongly correlates with blood NfL (Disanto et al. 2017; Hansson et al. 2017; Kuhle et al. 2016; Marques et al. 2019; Novakova et al. 2017), including in a cohort of mainly untreated PWH (Gisslén et al. 2015). However, whether this strong correlation persists in PWH on virally suppressive ART is unknown. Once NfL is released from CNS neuro-axonal units, it reaches the interstitial fluid which communicates freely with CSF and blood. While it could be postulated that an impaired blood–brain barrier (from untreated HIV-disease or alcohol and recreational drug use) may be associated with higher transfer of NfL from the CSF into the bloodstream, several studies have demonstrated that blood NfL concentration is not influenced by blood–brain barrier permeability (Gisslén et al. 2015; Kalm et al. 2017).

Our primary aim was to assess the correlation between CSF and plasma NfL in PWH on virally suppressive ART and in lifestyle-similar HIV-negative individuals. Secondly, we aimed to determine factors associated with CSF and plasma NfL in PWH on virally suppressive ART and lifestyle-similar HIV-negative individuals.

## Methods

### Participants

The ‘COmorBidity in Relation to AIDS’ (COBRA) cohort was a study of PWH aged ≥ 45 years, with viral suppression on ART for ≥ 12 months, recruited between January 2013 and October 2014 at two large HIV treatment centres in Amsterdam, The Netherlands and London, UK (De Francesco et al. 2018). Age- and lifestyle-similar HIV-negative individuals were recruited at sexual health clinics and community groups.

For this sub-study, a stored plasma sample from each participant’s baseline visit was retrieved. Participant demographics, medical history, lifestyle details and biological samples (CSF and blood) were obtained within the context of the study protocol and all participants gave written informed consent for future use of their stored data and samples.

Additionally, blood-based materials from 35 age-matched unselected blood bank donors were obtained from the Dutch national blood bank in Amsterdam, The Netherlands (www.sanquin.nl), as part of a separate sub-study within the main COBRA study (Booiman et al. 2017). Age and gender information were available for the blood bank donors. Blood bank donors from The Netherlands are routinely screened for blood-borne infections such as HIV, HTLV, hepatitis B, hepatitis C and syphilis. Individuals over the age of 70 years or those with high-risk behaviour for acquiring blood borne infections are excluded from blood donation. Overall, blood bank donors are at lower risk of acquiring blood-borne infections than the general population, but are often used as a control group for comparative studies.

I retrieved stored plasma samples from the blood bank donors who were included in the previous COBRA sub-study, and measured plasma NfL using the same technique as described below.

### NfL measurement

CSF NfL was measured previously in the COBRA study, using sandwich ELISA (NF-light ELISA kit; UmanDiagnostics AB, Umeå, Sweden) (De Francesco et al. 2018). For this sub-study, plasma NfL was measured using a NF-light digital immunoassay on the Simoa HD-1 platform (Quanterix Corp., Boston, MA, USA), at the UK Dementia Research Institute, University College London, UK (Hendricks et al. 2019). The lower limit of quantification (LLOQ) determined by the manufacturer is 0.174 pg/mL. Samples were analysed in duplicate, diluted 1:4 and results were all above the LLOQ. Analyses were performed by the same technician using a single batch of reagents. The intra- and inter-assay coefficients of variations were below 6% and 15%, respectively.

### Other laboratory measurements

Laboratory parameters measured previously in the COBRA study included blood (CD4^+^ and CD8^+^ counts, neopterin, albumin, creatinine and HIV RNA) and CSF (neopterin, albumin and HIV RNA). Blood and CSF HIV RNA were measured using the Abbott RealTime M2000 assay (Abbot, Chicago, USA) with a lower limit of detection of 40 copies/mL. Estimated glomerular filtration rate was calculated using the Chronic Kidney Disease Epidemiology Collaboration (CKD-EPI) formula (Levey et al. 2009).

### Statistical analyses

Statistical analyses were performed using SAS version 9.4 (SAS Institute, Cary, NC, USA). P values < 0.05 were considered statistically significant throughout. Differences in CSF and plasma NfL concentrations between PWH, HIV-negative participants and blood bank donors (where applicable) were tested for significance using Wilcoxon’s test. The correlation between CSF and plasma NfL (after log10-transformation) was assessed using Pearson’s correlation coefficient and the difference between groups was tested for significance using the Z-test after Fisher’s transformation. Log_10_-transformed CSF and plasma NfL Z-scores were calculated to enable direct comparisons between CSF and plasma NfL. A Bland–Altman graph (Bland and Altman 1999) was plotted to describe the agreement between log_10_-transformed CSF and plasma NfL Z-scores. This represents a plot of the difference between the CSF and plasma NfL Z-score against their average for each individual. Linear regression models were used to identify factors independently associated with log_10_-transformed CSF and plasma NfL, standardised to Z-scores with a mean of 0 and standard deviation of 1, separately in PWH and HIV-negative participants. Factors associated with either CSF or plasma NfL in either PWH or HIV-negative participants in univariable analyses, were included in multivariable models. Factors included in the univariable linear regression analysis were female gender, age, Black African ethnicity, men who have sex with men sexual orientation, weight, on hypertensive medication, body mass index, current tobacco smoker, ex-tobacco smoker, current alcohol consumption, past alcohol consumption, recent recreational drug use, ever used injected recreational drugs, global cognitive function T-score, current CD4^+^ and CD8^+^ T cell counts, current CD4^+^/8^+^ ratio, serum creatinine, serum albumin, cerebrospinal fluid protein, cerebrospinal fluid neopterin and plasma neopterin. HIV-specific parameters significantly associated in univariable analyses were added to an additional model fitted in PWH only. The HIV-specific factors included in the univariable linear regression analysis included time since HIV diagnosis, time on ART, nadir CD4^+^ count, years with CD4^+^ count <200 cells/µL, prior AIDS diagnosis, on a non-nucleoside reverse transcriptase inhibitor (NNRTI), on a protease inhibitor (PI), on tenofovir-disoproxil fumarate (TDF) and on atazanavir.

## Results

Of the COBRA participants, 132/134 PWH and 79/79 HIV-negative participants and all 35 blood bank donors who were included in the previous COBRA sub-study had sufficient stored plasma available and were included in this analysis. Socio-demographic, lifestyle characteristics and anthropometrics at study baseline were described previously (De Francesco et al. 2018). Briefly, PWH and HIV-negative participants in this sub-study were similar in terms of age, gender, sexual orientation, anthropometric measurements, years of education and lifestyle factors. PWH were less likely to be of white ethnicity (p = 0.02) and current alcohol drinkers (p = 0.01) than HIV-negative participants (Table 1).

**Table 1:** Sociodemographic, anthropometric, lifestyle and HIV-related characteristics of study participants

**Variable, *n* (%) or median (IQR)**	**PWH** **(*N* = 132)**	**HIV-negative participants (*N* = 79)**	***p* value**
Gender			0.66
Female	8 (6.1%)	6 (7.6%)	
Male	124 (93.9%)	73 (92.4%)	
Ethnicity			0.02
Black-African	16 (12.2%)	2 (2.6%)	
White	115 (87.8%)	76 (97.4%)	
Sexual orientation			0.39
Men who have sex with men	103 (78.0%)	59 (74.7%)	
Bisexual	10 (7.6%)	4 (5.1%)	
Heterosexual	18 (13.6%)	16 (20.2%)	
Age, years	56 (51, 62)	57 (52, 64)	0.30
Years of education	14 (13, 16)	16 (14, 17)	0.21
Weight, kg	79.6 (70.0, 87.7)	81.2 (72.5, 91.0)	0.14
BMI, kg/m^2^	24.7 (22.7, 27.5)	24.6 (23.2, 28.4)	0.35
Current smoker	39 (29.6%)	20 (25.3%)	0.22
Years of smoking (current/ex-smokers)	29 (19, 37)	26 (15, 39)	0.40
Current alcohol drinker	103 (78.0%)	71 (91.0%)	0.04
Years of drinking (current/ex drinkers)	29 (19, 37)	41 (35, 47)	0.01
Alcohol units/week (current drinkers)	5.5 (1.5, 15.0)	7.5 (1.5, 17.5)	0.39
Ever used injected drugs	5 (3.8%)	0 (0.0%)	0.16
Use of recreational drugs in past 6 months	43 (32.6%)	18 (22.8%)	0.13
On hypertensive medication	29 (22.0%)	14 (17.7%)	0.46
CSF albumin, mg/L	299 (210, 368)	267 (214, 326)	0.43
Serum albumin, g/L	43 (41, 46)	43 (40, 45)	0.08
CSF:serum albumin	6.9 (4.9, 8.5)	6.2 (5.0, 7.5)	0.38
Creatinine, mmol/L	85.5 (76, 100)	79 (72, 92)	
eGFR, mL/min per 1.73 m^2^	85.5 (71.0, 97.3)	92.0 (77.4, 102.3)	0.03
Years since HIV diagnosis	15.0 (9.0, 19.6)	N/A	N/A
Duration of ART, years	12.8 (7.8, 16.9)	N/A	N/A
CSF HIV RNA viral load <40 copies/mL	130 (98.5%)	N/A	N/A
CD4^+^/CD8^+^ T cell count ratio	0.83 (0.60, 1.11)	2.00 (1.44, 2.65)	N/A
Prior AIDS event	42 (31.8%)	N/A	N/A
CD4^+^ T cell count [cells/µL]	618 (472, 809)	900 (692, 1174)	N/A
Nadir CD4^+^ T cell count [cells/µL]	175 (85, 240)	N/A	N/A
Months with CD4^+^ T cell count < 200 cells/µL	1.6 (0.0, 9.8)	N/A	N/A

*PWH* people with HIV, *BMI* body mass index, *CSF* cerebrospinal fluid, *eGFR* estimated glomerular filtration rate, *ART* antiretroviral treament

Blood bank donors were similar in age (median (IQR) 59 years (52, 65)) to the COBRA participants (p = 0.41), but a higher proportion of blood bank donors were female (48.6%), compared to the COBRA cohort (p < 0.001).

PWH had been diagnosed for a median (IQR) of 15.0 (9.0, 19.6) years with a median (IQR) current CD4^+^ count of 618 (472, 809) cells/µL, current CD4:CD8 ratio of 0.83 (0.60, 1.11) and a nadir CD4^+^ count of 175 (85, 240) cells/µL. PWH had been on ART for a median (IQR) of 12.8 (7.8, 16.9) years, all PWH had undetectable plasma HIV RNA and 130/132 had undetectable CSF HIV RNA (Table 1).

Median (IQR) CSF:serum albumin ratio (a marker of blood–brain barrier dysfunction) was similar between the groups (6.9 (4.9, 8.5) and 6.2 (5.0, 7.5) in PWH and HIV-negative participants, respectively). Serum creatinine was significantly higher in PWH, median (IQR) 85.5 (76, 100) vs 79 (72, 92) µmol/L in PWH and HIV-negative participants respectively, p = 0.03 (Table 1).

### CSF and plasma NfL results

**Fig. 1:**
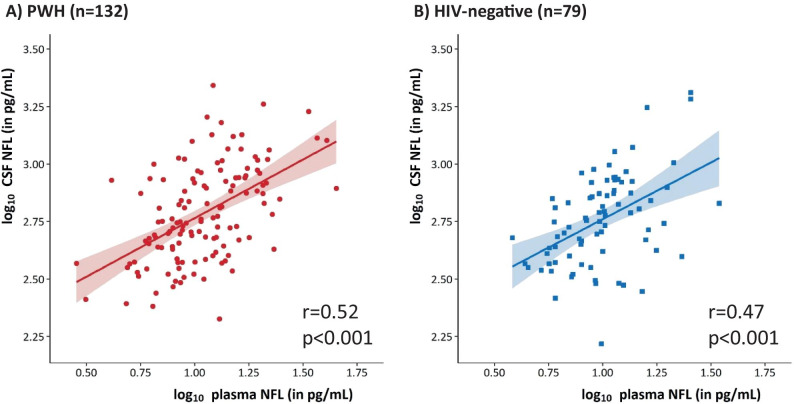
Correlation between log-transformed plasma and CSF NFL concentration in PWH (**A**) and HIV-negative controls (**B**)

Comparing PWH and HIV-negative participants, neither CSF NfL (median (IQR) 570 (430, 864) vs 568 (408, 767) pg/mL, p = 0.37), nor plasma NfL (median (IQR) 10.7 (7.9, 14.9) vs 9.9 (7.2, 12.5) pg/mL, p = 0.15) differed significantly between the groups. The correlation between CSF and plasma NfL did not differ significantly by HIV status (PWH: r = 0.52 (95% confidence interval (CI) 0.38–0.63); HIV-negative participants: r = 0.47 (95%CI 0.27–0.62), p value testing the difference = 0.63) (Fig. 1).

Plasma NfL was significantly lower in the blood bank donors cohort (median (IQR) 6.15 (4.94, 7.15)) compared to people with HIV (p < 0.001) and HIV-negative participants (p < 0.001).

**Fig. 2:**
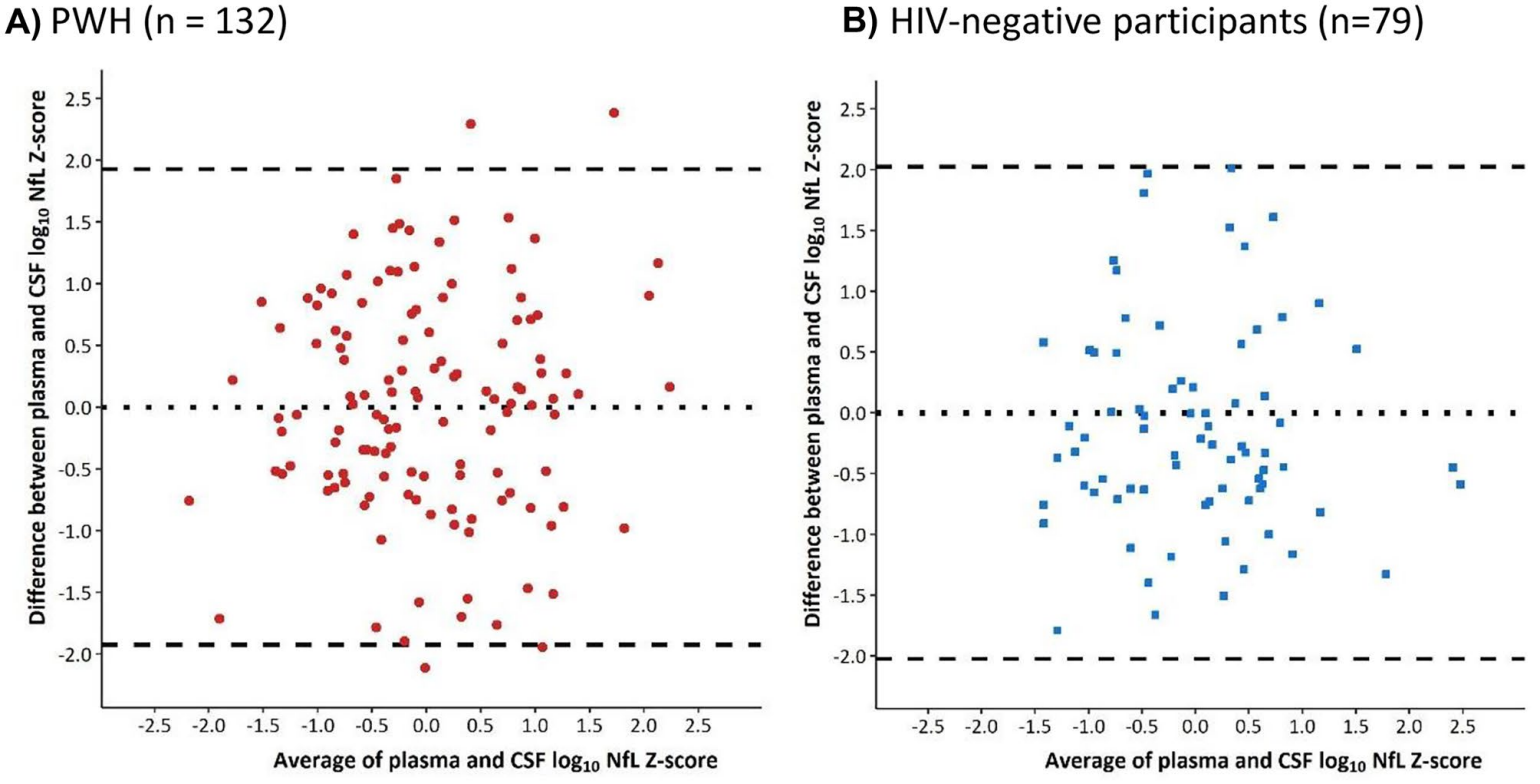
Bland–Altman plot of plasma and CSF NFL *Z*-scores in PWH (**A**) and HIV-negative participants (**B**)

Bland–Altman plots are reported in Fig. 2. Among PWH, there was not a systematic over- or under-estimation of one score compared to the other. Mean (95%CI) difference between the scores was 0.00 (−0.17, 0.17), p = 0.99 and the agreement between the two scores was not altered depending on the average CSF and plasma NfL Z-score; the correlation (95%CI) between the difference and the mean of the two scores was −0.004 (−0.18, 0.17), p = 0.96. The lower and upper limits of agreement (95%CI) between the two measurements were −1.97 (−2.25, −1.68) and 1.97 (1.68, 2.25), respectively, indicating substantial discrepancies between the NfL Z-scores obtained from the two compartments. A similar pattern was observed among HIV-negative individuals, with a mean (95%CI) difference of 0.00 (−0.23, 0.23) and no correlation between the difference and average [r (95%CI) = 0.00 (−0.22, 0.22), p = 0.99].

### Factors associated with CSF and plasma NfL

In multivariable regression analysis, higher CSF NfL Z-score was associated with older age and higher CSF protein in both PWH and HIV-negative participants; lower CSF NfL Z-score was significantly associated with female gender in HIV-negative participants (0.83 log_10_ CSF NfL Z-score lower compared to men, p = 0.03) and higher CSF NfL Z-score approached a statistically significant association with lower bodyweight in PWH (0.05 log_10_ CSF NfL Z-score higher per 5 kg decrease in bodyweight, p = 0.05) (Table 2). Higher plasma NfL Z-score was associated with older age, higher serum creatinine and lower bodyweight in both PWH and HIV-negative participants, and higher CSF NfL Z-score approached a statistically significant association with lower serum albumin in PWH (0.31 log_10_ CSF NfL Z-score higher per 10 g/L decrease in serum albumin, p = 0.05) (Table 2).

**Table 2:** Multivariable models to compare factors associated with CSF and plasma log_10_ NfL *Z*-scores between PWH and HIV-negative participants, excluding HIV-specific parameters^a,b^

	**Variable**	**PWH**		**HIV-negative controls**	
		**Change (95% CI)**	***p* value**	**Change (95% CI)**	***p* value**
**log**_**10**_** CSF NFL *Z*-score**	Female vs male	−0.44 (−1.05, 0.17)	0.15	−0.83 (−1.56, −0.10)	0.03
	Age (per 10 years)	0.69 (0.50, 0.88)	< 0.001	0.74 (0.46, 1.02)	< 0.001
	Weight (per 5 kg)	−0.05 (−0.10, 0.00)	0.05	0.02 (−0.04, 0.07)	0.55
	Hypertensive medication (yes vs no)	0.26 (−0.08, 0.61)	0.13	−0.05 (−0.64, 0.55)	0.87
	Serum creatinine (per mg/dL)	0.47 (−0.29, 1.24)	0.22	−0.71 (−2.00, 0.59)	0.28
	Serum albumin (per 10 g/L)	0.00 (−0.33, 0.34)	0.98	0.09 (−0.50, 0.67)	0.77
	CSF protein (per g/L)	1.46 (0.63, 2.29)	< 0.001	1.81 (0.37, 3.25)	0.01
	CSF neopterin	−0.01 (−0.04, 0.01)	0.33	0.04 (−0.06, 0.13)	0.45
	Plasma neopterin	0.02 (−0.01, 0.05)	0.18	−0.002 (−0.11, 0.11)	0.96
**log**_**10**_** plasma NFL *Z*-score**	Female vs male	0.13 (−0.44, 0.69)	0.66	0.57 (−0.13, 1.27)	0.11
	Age (per 10 years)	0.62 (0.44, 0.80)	< 0.001	0.63 (0.36, 0.90)	< 0.001
	Weight (per 5 kg)	−0.13 (−0.18, −0.08)	< 0.001	−0.06 (−0.11, 0.00)	0.05
	Hypertensive medication (yes vs no)	0.28 (−0.03, 0.60)	0.08	0.18 (−0.39, 0.76)	0.53
	Serum creatinine (per mg/dL)	0.73 (0.02, 1.44)	0.04	1.64 (0.39, 2.89)	0.01
	Serum albumin (per 10 g/L)	−0.31 (−0.63, 0.00)	0.05	−0.39 (−0.95, 0.18)	0.18
	CSF protein (per g/L)	−0.30 (−1.07, 0.47)	0.44	0.45 (−0.94, 1.84)	0.52
	CSF neopterin	0.01 (−0.02, 0.03)	0.69	0.08 (−0.01, 0.17)	0.07
	Plasma neopterin	0.03 (0.00, 0.05)	0.07	0.01 (−0.09, 0.11)	0.85

^a^Parameter estimates reflect the associated impact (measured in standard deviations) of each independent variable in the model on the dependent variable

^b^Variables included in the univariable linear regression analysis that were not statistically significant and not included in the multivariable model were Black African ethnicity, men who have sex with men sexual orientation, body mass index, current tobacco smoker, ex-tobacco smoker, current alcohol consumption, past alcohol consumption, recent recreational drug use, ever used injected recreational drugs, global cognitive function *T*-score, current CD4^+^ and CD8^+^ count and current CD4^+^/8^+^ ratio

Among PWH, none of the HIV-specific parameters showed a significant association with either CSF and plasma NfL Z-scores, after accounting for other factors (Table 3).

**Table 3:** Multivariable models to assess factors associated with CSF and plasma log_10_ NFL Z-scores in PWH only, and including HIV-related parameters^a,b^

	**Variable**	**PWH**	
		**Change (95% CI)**	***p* value**
**log**_**10**_** CSF NFL *Z*-score**	Female vs male	−0.48 (−1.09, 0.14)	0.13
	Age (per 10 years)	0.69 (0.49, 0.89)	< 0.001
	Weight (per 5 kg)	−0.06 (−0.11, −0.01)	0.03
	Hypertensive medication (yes vs no)	0.28 (−0.06, 0.63)	0.11
	Serum creatinine (per mg/dL)	0.41 (−0.36, 1.18)	0.30
	Serum albumin (per 10 g/L)	−0.01 (−0.35, 0.33)	0.96
	CSF protein (per g/L)	1.37 (0.53, 2.21)	0.002
	CSF neopterin	−0.01 (−0.04, 0.01)	0.34
	Plasma neopterin	0.02 (−0.01, 0.05)	0.22
	Time since HIV diagnosis (years)	0.18 (−0.22, 0.59)	0.38
	Time on cART (years)	−0.35 (−0.81, 0.11)	0.14
**log**_**10**_** plasma NFL *Z*-score**	Female vs male	0.18 (−0.38, 0.75)	0.52
	Age (per 10 years)	0.58 (0.39, 0.76)	< 0.001
	Weight (per 5 kg)	−0.12 (−0.17, −0.07)	< 0.001
	Hypertensive medication (yes vs no)	0.25 (−0.07, 0.57)	0.12
	Serum creatinine (per mg/dL)	0.79 (0.08, 1.50)	0.03
	Serum albumin (per 10 g/L)	−0.29 (−0.60, 0.02)	0.07
	CSF protein (per g/L)	−0.23 (−1.00, 0.55)	0.56
	CSF neopterin	0.01 (−0.02, 0.03)	0.53
	Plasma neopterin	0.03 (0.00, 0.06)	0.05
	Time since HIV diagnosis (years)	0.30 (−0.08, 0.67)	0.12
	Time on cART (years)	−0.17 (−0.59, 0.26)	0.44

^a^Parameter estimates reflect the associated impact (measured in standard deviations) of each independent variable in the model on the dependent variable

^b^Variables included in the univariable linear regression analysis that were not statistically significant and not included in the multivariable model were Black African ethnicity, men who have sex with men sexual orientation, body mass index, current tobacco smoker, ex-tobacco smoker, current alcohol consumption, past alcohol consumption, recent recreational drug use, ever used injected recreational drugs, global cognitive function *T*-score, current CD4^+^ and CD8^+^ count, current CD4^+^/8^+^ ratio, nadir CD4^+^ count, years with CD4^+^ count < 200 cells/µL, prior AIDS diagnosis, on a non-nucleoside reverse transcriptase inhibitor (NNRTI), on a protease inhibitor (PI), on tenofovir-disoproxil fumarate (TDF) and on atazanavir

## Discussion

In this study, we observed that the correlation between CSF and plasma NfL in PWH is moderate and similar to that observed in a cohort of lifestyle-similar HIV-negative controls. The previous report suggesting a strong correlation in a cohort of mainly untreated PWH (Gisslén et al. 2015) is likely a consequence of the much higher concentrations of CSF and plasma NfL seen in that cohort, where the median CSF and plasma NfL concentrations in individuals with HIV-associated dementia were 16,185 pg/mL and 114 pg/mL, respectively.

Concentrations of CSF and plasma NfL were similar in PWH and HIV-negative participants, suggesting that PWH on ART do not have increased biomarker evidence of neuro-axonal injury compared to lifestyle-similar HIV-negative individuals. CSF/plasma NfL ratios also did not differ by HIV status, with plasma NfL being 55–65 times lower compared to CSF NfL, consistent with published literature (Gisslén et al. 2015). In view of the lack of significant difference in cerebrospinal fluid Nfl, plasma NfL and cerebrospinal fluid/plasma NfL ratios between PWH on suppressive ART and lifestyle-similar HIV-negative participants, we did not correlate our findings with the cerebral imaging data from this cohort, given that these data have already been previously reported.

Our results demonstrated that plasma NfL concentration was significantly lower in the blood bank donors compared to the PWH and HIV-negative controls enrolled into the COBRA study. Given the lack of additional demographic factors apart from age and gender, and concurrent cerebrospinal fluid samples, we were unable to characterise this further, and can only conclude that based on the limited information available, PWH and HIV-negative controls in the COBRA study were observed to have evidence of higher plasma NfL concentrations as a biomarker of neuro-axonal injury, compared to blood bank donors.

Our results demonstrating that higher CSF and plasma NfL were associated with older age echoes the findings in published studies (Disanto et al. 2017; Yilmaz et al. 2017). The finding that higher CSF NfL is associated with higher CSF protein is unsurprising, given that higher CSF protein may indicate CNS pathology, and hence higher neuronal damage. Our finding that female gender is associated with lower cerebrospinal fluid NfL is not wholly unexpected, though the published data in this regard remains controversial with some studies demonstrating that cerebrospinal fluid NfL is higher in males (Forgrave et al. 2019; Lin et al. 2018), higher in females (Lu et al. 2015) or not significantly different by gender (Manouchehrinia et al. 2020a,  b).

Our results suggest that consideration of renal function and body composition is an important requirement when interpreting plasma NfL. While renal excretion of NfL has not been extensively described, NfL has a molecular weight of about 68 kDa and a high abundance of negatively charged amino acids in its sequence, both of which theoretically decrease its ability to cross the glomerular filtration barrier. Published data demonstrate that while plasma NfL decreased after switching from tenofovir disoproxil fumarate to tenofovir alafenamide fumarate, no significant correlations between plasma NfL and serum creatinine pre- and post-switch, or between changes in plasma NfL and serum creatinine pre- and post-switch were observed (Hermansson et al. 2019). Although lower bodyweight was independently associated with higher plasma NfL, body mass index was not an associated factor in our study, in contrast to a publication demonstrating a negative association between plasma NfL and body mass index (Manouchehrinia et al. 2020a,  b).

Strengths of our study include the comprehensively-phenotyped cohort and the lifestyle-similar HIV-negative individuals. Limitations include the COBRA participants being predominantly middle-aged white men who have sex with men from Western Europe, thus more data on the correlation between CSF and plasma NFL are required from other populations, as it is unknown whether our findings can be extrapolated to other populations. Further work will need to be performed to assess plasma NfL in younger PWH who may not have been on suppressive ART for as long as the COBRA study participants. Another limitation was the lack of data on other factors that may increase plasma NFL (e.g. peripheral neuropathy) and concomitant neurotoxic drug use. The number of female participants in our study was small and studies involving more female participants would be needed to verify the association between female gender and lower CSF NfL.

Our results demonstrate that cerebrospinal fluid and plasma NfL concentrations are similar in people with HIV on suppressive antiretroviral treatment and in lifestyle-similar HIV-negative individuals. Furthermore, our findings suggest that plasma NfL may be a promising and more easily accessible surrogate biomarker of neuronal integrity. Cerebrospinal fluid NfL measurements closely reflect processes taking place within the CNS, whereas both the CNS and PNS contribute to plasma NfL measurements, which may lead to wider variations in plasma NfL measurements. In view of this, further work is required to improve our understanding of the factors that influence plasma NfL concentrations.

## References

[CR1] Abdulle S, Mellgren Å, Brew BJ, Cinque P, Hagberg L, Price RW, Rosengren L, Gisslén M (2007). CSF neurofilament protein (NFL) - A marker of active HIV-related neurodegeneration. J Neurol.

[CR2] Bland JM, Altman DG (1999). Measuring agreement in method comparison studies. Stat Methods Med Res.

[CR3] Booiman T, Wit FW, Girigorie AF, Maurer I, De Francesco D, Sabin CA, Harskamp AM, Prins M, Franceschi C, Deeks SG, Winston A, Reiss P, Kootstra NA (2017). Terminal differentiation of T cells is strongly associated with CMV infection and increased in HIV-positive individuals on ART and lifestyle matched controls. PLoS ONE.

[CR4] Bridel C, Van Wieringen WN, Zetterberg H, Tijms BM, Teunissen CE, Alvarez-Cermeño JC, Andreasson U, Axelsson M, Bäckström DC, Bartos A, Bjerke M, Blennow K, Boxer A, Brundin L, Burman J, Christensen T, Fialová L, Forsgren L, Frederiksen JL, Wild EJ (2019). Diagnostic Value of Cerebrospinal Fluid Neurofilament Light Protein in Neurology: A Systematic Review and Meta-analysis. JAMA Neurol.

[CR5] De Francesco D, Wit FW, Cole JH, Kootstra NA, Winston A, Sabin CA, Underwood J, Van Zoest RA, Schouten J, Kooij KW, Prins M, Guaraldi G, Caan MWA, Burger D, Franceschi C, Libert C, Bü Rkle A, Reiss P (2018). The “COmorBidity in Relation to AIDS” (COBRA) cohort: Design, methods and participant characteristics, on behalf of the COmorBidity in Relation to AIDS (COBRA) collaboration. PLoS ONE.

[CR6] Disanto G, Barro C, Benkert P, Naegelin Y, Schädelin S, Giardiello A, Zecca C, Blennow K, Zetterberg H, Leppert D, Kappos L, Gobbi C, Kuhle J (2017). Serum Neurofilament light: A biomarker of neuronal damage in multiple sclerosis. Ann Neurol.

[CR7] Forgrave LM, Ma M, Best JR, DeMarco ML (2019). The diagnostic performance of neurofilament light chain in CSF and blood for Alzheimer’s disease, frontotemporal dementia, and amyotrophic lateral sclerosis: a systematic review and meta-analysis. In Alzheimer’s and Dementia: Diagnosis, Assessment and Disease Monitoring.

[CR8] Gaetani L, Blennow K, Calabresi P, Di Filippo M, Parnetti L, Zetterberg H (2019). Neurofilament light chain as a biomarker in neurological disorders. In Journal of Neurology, Neurosurgery and Psychiatry.

[CR9] Gisslén M, Hagberg L, Brew BJ, Cinque P, Price RW, Rosengren L (2007). Elevated Cerebrospinal Fluid Neurofilament Light Protein Concentrations Predict the Development of AIDS Dementia Complex. J Infect Dis.

[CR10] Gisslén M, Price RW, Andreasson U, Norgren N, Nilsson S, Hagberg L, Fuchs D, Spudich S, Blennow K, Zetterberg H (2015). Plasma concentration of the neurofilament light protein (NFL) is a biomarker of CNS injury in hiv infection: a cross-sectional study. EBioMedicine.

[CR11] Hansson O, Janelidze S, Hall S, Magdalinou N, Lees AJ, Andreasson U, Norgren N, Linder J, Forsgren L, Constantinescu R, Zetterberg H, Blennow K (2017). Blood-based NfL: a biomarker for differential diagnosis of parkinsonian disorder. Neurology.

[CR12] Hendricks R, Baker D, Brumm J, Davancaze T, Harp C, Herman A, Von Büdingen HC, Townsend M, Fischer SK (2019). Establishment of neurofilament light chain Simoa assay in cerebrospinal fluid and blood. Bioanalysis.

[CR13] Hermansson L, Yilmaz A, Price RW, Nilsson S, McCallister S, Makadzange T, Das M, Zetterberg H, Blennow K, Gisslen M (2019). Plasma concentration of neurofilament light chain protein decreases after switching from tenofovir disoproxil fumarate to tenofovir alafenamide fumarate. PLoS ONE.

[CR14] Kalm M, Boström M, Sandelius Å, Eriksson Y, Ek CJ, Blennow K, Björk-Eriksson T, Zetterberg H (2017). Serum concentrations of the axonal injury marker neurofilament light protein are not influenced by blood-brain barrier permeability. Brain Res.

[CR15] Khalil M, Teunissen CE, Otto M, Piehl F, Sormani MP, Gattringer T, Barro C, Kappos L, Comabella M, Fazekas F, Petzold A, Blennow K, Zetterberg H, Kuhle J (2018). Neurofilaments as biomarkers in neurological disorders. In Nature Reviews Neurology.

[CR16] Krut JJ, Mellberg T, Price RW, Hagberg L, Fuchs D, Rosengren L, Nilsson S, Zetterberg H, Gissl NM (2014) Biomarker evidence of axonal injury in neuroasymptomatic HIV-1 patients. PLoS One 9(2). 10.1371/journal.pone.0088591 10.1371/journal.pone.0088591 PMC3921217 24523921

[CR17] Kuhle J, Barro C, Andreasson U, Derfuss T, Lindberg R, Sandelius Å, Liman V, Norgren N, Blennow K, Zetterberg H (2016). Comparison of three analytical platforms for quantification of the neurofilament light chain in blood samples: ELISA, electrochemiluminescence immunoassay and Simoa. Clin Chem Lab Med.

[CR18] Levey AS, Stevens LA, Schmid CH, Zhang Y, Castro AF, Feldman HI, Kusek JW, Eggers P, Lente FV, Greene T, Coresh J (2009). A new equation to estimate glomerular filtration rate. Ann Intern Med.

[CR19] Lin YS, Lee WJ, Wang SJ, Fuh JL (2018). Levels of plasma neurofilament light chain and cognitive function in patients with Alzheimer or Parkinson disease. Sci Rep.

[CR20] Lu C-H, Macdonald-Wallis C, Gray E, Pearce N, Petzold A, Norgren N, Giovannoni G, Fratta P, Sidle K, Fish M, Orrell R, Howard R, Talbot K, Greensmith L, Kuhle J, Turner MR, Malaspina A (2015). Neurofilament light chain: a prognostic biomarker in amyotrophic lateral sclerosis. Neurology.

[CR21] Manouchehrinia A, Piehl F, Hillert J, Kuhle J, Alfredsson L, Olsson T, Kockum I (2020). Confounding effect of blood volume and body mass index on blood neurofilament light chain levels. Annals of Clinical and Translational Neurology.

[CR22] Manouchehrinia A, Stridh P, Khademi M, Leppert D, Barro C, Michalak Z, Benkert P, Lycke J, Alfredsson L, Kappos L, Piehl F, Olsson T, Kuhle J, Kockum I (2020). Plasma neurofilament light levels are associated with risk of disability in multiple sclerosis. Neurology.

[CR23] Marques TM, Van Rumund A, Oeckl P, Kuiperij HB, Esselink RAJ, Bloem BR, Otto M, Verbeek MM (2019). Serum NFL discriminates Parkinson disease from atypical parkinsonisms. Neurology.

[CR24] Mellgren Å, Price RW, Hagberg L, Rosengren L, Brew BJ, Gisslén M (2007). Antiretroviral treatment reduces increased CSF neurofilament protein (NFL) in HIV-1 infection. Neurology.

[CR25] Novakova L, Zetterberg H, Sundström P, Axelsson M, Khademi M, Gunnarsson M, Malmeström C, Svenningsson A, Olsson T, Piehl F, Blennow K, Lycke J (2017). Monitoring disease activity in multiple sclerosis using serum neurofilament light protein. Neurology.

[CR26] Olsson B, Portelius E, Cullen NC, Sandelius Å, Zetterberg H, Andreasson U, Höglund K, Irwin DJ, Grossman M, Weintraub D, Chen-Plotkin A, Wolk D, McCluskey L, Elman L, Shaw LM, Toledo JB, McBride J, Hernandez-Con P, Lee VMY, Blennow K (2019). Association of Cerebrospinal Fluid Neurofilament Light Protein Levels with Cognition in Patients with Dementia, Motor Neuron Disease, and Movement Disorders. JAMA Neurol.

[CR27] Peterson J, Gisslen M, Zetterberg H, Fuchs D, Shacklett BL, Hagberg L, Yiannoutsos CT, Spudich SS, Price RW (2014). Cerebrospinal fluid (CSF) neuronal biomarkers across the spectrum of HIV infection: Hierarchy of injury and detection. PLoS ONE.

[CR28] Yilmaz A, Blennow K, Hagberg L, Nilsson S, Price RW, Schouten J, Spudich S, Underwood J, Zetterberg H, Gisslén M (2017) Neurofilament light chain protein as a marker of neuronal injury: review of its use in HIV-1 infection and reference values for HIV-negative controls. In Expert Review of Molecular Diagnostics 17(8)761–770. 10.1080/14737159.2017.1341313 10.1080/14737159.2017.1341313 28598205

[CR29] Zetterberg H (2016). Neurofilament light: a dynamic cross-disease fluid biomarker for neurodegeneration. In Neuron.

